# Male Fertility under Environmental Stress: Do Polyamines Act as Pollen Tube Growth Protectants?

**DOI:** 10.3390/ijms23031874

**Published:** 2022-02-07

**Authors:** Iris Aloisi, Chiara Piccini, Giampiero Cai, Stefano Del Duca

**Affiliations:** 1 Dipartimento di Scienze Biologiche, Geologiche e Ambientali, Università Degli Studi di Bologna, Via Irnerio, 40126 Bologna, Italy; iris.aloisi2@unibo.it (I.A.); stefano.delduca@unibo.it (S.D.D.); 2 Dipartimento di Scienze della Vita, University of Siena, Via Mattioli 4, 53100 Siena, Italy; piccini3@student.unisi.it; 3 Interdepartmental Center for Agri-Food Industrial Research, University of Bologna, 40126 Bologna, Italy

**Keywords:** plant reproduction, pollen tube growth, environmental stress, polyamines

## Abstract

Although pollen structure and morphology evolved toward the optimization of stability and fertilization efficiency, its performance is affected by harsh environmental conditions, e.g., heat, cold, drought, pollutants, and other stressors. These phenomena are expected to increase in the coming years in relation to predicted environmental scenarios, contributing to a rapid increase in the interest of the scientific community in understanding the molecular and physiological responses implemented by male gametophyte to accomplish reproduction. Here, after a brief introduction summarizing the main events underlying pollen physiology with a focus on polyamine involvement in its development and germination, we review the main effects that environmental stresses can cause on pollen. We report the most relevant evidence in the literature underlying morphological, cytoskeletal, metabolic and signaling alterations involved in stress perception and response, focusing on the final stage of pollen life, i.e., from when it hydrates, to pollen tube growth and sperm cell transport, with these being the most sensitive to environmental changes. Finally, we hypothesize the molecular mechanisms through which polyamines, well-known molecules involved in plant development, stress response and adaptation, can exert a protective action against environmental stresses in pollen by decoding the essential steps and the intersection between polyamines and pollen tube growth mechanisms.

## 1. Polyamines in Pollen Development

**Figure 1:**
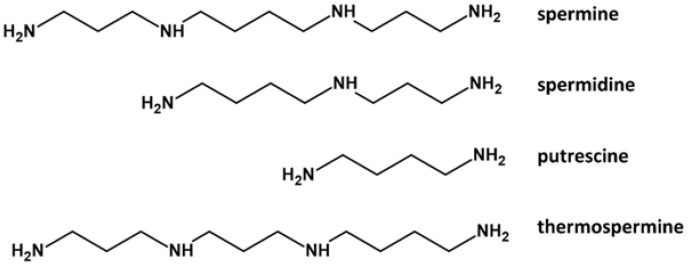
The main Pas identified in plant reproductive organs. Molecules were drawn using ACD/ChemSketch Freeware software (https://www.acdlabs.com/index.php, accessed on 4 January 2022).

Polyamines (PAs), i.e., spermine, spermidine, and putrescine (Figure 1) are small organic polycations with a widespread presence in all living organisms [1]. Another tetra-amine, i.e., thermospermine, has been detected in archaea, diatoms, and plants, but not in animals or bacteria [2]. PAs in plants are involved in many processes, such as organogenesis, embryogenesis, floral and fruit development, leaf senescence, and plant abiotic and biotic stress responses. PAs are also highly critical in the process of plant reproduction, from pollen development to fertilization [3,4] to self-incompatibility [5]. In cells, their concentration depends on the balance among biosynthesis, degradation, and transport [6]. More generally, PAs regulate plant cell growth and are involved in external stimuli perception and in counteracting adverse environmental conditions [7,8,9]. Changes in plant PA metabolism occur in response to a variety of abiotic and biotic stresses [10]; their levels can increase dramatically, and for example, putrescine can reach up to 1.2% of dry matter, or approximately 20% of total nitrogen in stressed plants. Pas can act as cellular signals in a crosstalk with hormonal pathways, including abscisic acid (ABA) to cope with abiotic stress [11]. Similar to other aliphatic Pas, a role in defense against stresses has also been proposed for thermospermine [12].

We recently reviewed the involvement of Pas during the main pollen developmental stages, a complex and well-coordinated process governed by genetic and enzymatic processes, some of which are modulated by Pas [3]. When pollen lands on the stigma of a receptive flower, it hydrates and produces a growing pollen tube to transport sperm cells and the vegetative nucleus. The pollen tube grows through the stigma and style following a precise set of extracellular signals [13,14], including PAs, which are released into the germination medium together with RNAs, neo-synthesized proteins, and the PAs cross-linking enzyme transglutaminase (TGase), suggesting their possible involvement in pollen tube/style adhesion [15]. Pollen tube growth occurs exclusively in the apical region through the accumulation of secretory vesicles carrying new cell wall material, new plasma membrane, and proteins [16]. Methyl-esterified pectins accumulate at the extreme apex of the pollen tube and are then converted to acidic pectins, thereby stiffening the cell wall by cross-linking with calcium ions. Subsequently, the cell wall is further strengthened by substantial deposition of callose and cellulose [17]. The process of cell wall deposition and modification depends on the control of vesicular secretion and in turn on a specific organization of the cytoskeleton. All these events rely on a central regulatory system based on membrane receptor proteins, GTPases, calcium ions, intracellular pH gradients, actin-binding proteins, as well as changes in the level of reactive oxygen species (ROS) and phosphoinositides (PI) [18,19,20,21,22,23]. PAs regulate all the above-mentioned aspects of pollen tube growth, as they take part in cell-wall structuring, Ca^2+^ and ROS-signaling as well as the organization of the cytoskeleton [3]. This regulatory system is the target of stressful conditions that can affect one or more molecular dowels that allow the pollen tube to grow.

As pollen and pollen tube growth are critically important in sexual plant reproduction, they have been the subject of a multitude of studies dealing with those environmental changes that can impair plant reproduction in both natural and anthropized areas [24,25]. Stress-induced effects include early or delayed flowering, asynchrony between male and female reproductive development, alteration and abnormal functioning of parental tissues, and defects in male and female gametes [26]. Pollen and pollen tubes are highly sensitive to stress conditions, sometimes more than the female gametophyte [27,28], and the impact of stress on pollen viability is well documented. Scientific research has focused on how abiotic stress causes pollen damage, how pollen implements tolerance mechanisms, and how pollen from different plant varieties or genotypes may differ in stress tolerance [29,30,31].

Here, we review recent evidence of how abiotic stress affects pollen performance. Considering that the topic is of great interest and is broad, we have made a careful selection of the bibliography. Therefore, not all recent bibliography has been cited, but only those closely related to the topic of the article. Finally, we discuss the outstanding issues and directions for future research that will further clarify our understanding of the involvement of PAs in overcoming stressful conditions.

## 2. Effects of Environmental Stress on Pollen Tube Growth

Any stress condition affecting pollen and the pollen tube can act at various levels, from cytological to biochemical to genetic. This is known already from the turn of the 1980s and 1990s, when pollen started to be analyzed for its responsiveness to stress conditions, showing the different response of distinct genotypes and proposing pollen for environmental monitoring [32,33,34]. Below, we report the main effects that environmental stresses can cause on pollen, categorized according to type of stress.

### 2.1. Heat Stress

Heat stress is probably the most studied stressful condition in pollen and plants in general. Investigation of pollen response to high temperatures started decades ago [35,36]; unfortunately, the knowledge about the mechanism of pollen tube growth was rather limited at that time and did not allow for the correlation between damage and cellular mechanisms. Currently, due to new evidence and abundant literature, pollen response to heat stress is much more understood both at the molecular and physiological levels. When released from anthers, pollen can be susceptible to heat stress, leading to partial or complete failure of reproduction. The effects of heat stress on pollen have been studied in several crop plants of agronomic interest, i.e., rice [28], sorghum [37], tomato [4], wheat [38], and maize [39], as well as in pea [30]. Plant models have also been investigated for deciphering pollen response to heat stress, i.e., *A. thaliana* [40] and tobacco [41]. In all cases, the studies focused on understanding how pollen might overcome the damage induced by heat stress. In addition, for crop plants, the selection of heat-resistant genotypes has also been undertaken. The large research interest in this field and the lack of standardized protocols for different plant species has recently led to a comprehensive review with suggested guidelines [25].

Pollen response to heat stress involves transcriptomic, proteomic, metabolomic and morphological alterations and results in reduced pollen performance. Pollen tube growth reduction might be explained by several pathways activated simultaneously after heat perception. While several heat shock protein genes (HSPs) and genes involved in defense responses are usually overexpressed and translated, a significant downregulation of genes encoding for proteins associated with growth and translation initiation was also demonstrated [39,40,41]. Moreover, genes involved in starch, hexose pools and fatty acids inter-conversion are strongly affected, resulting in reduced energy production [39] and possibly affecting pollen cell wall deposition. Heat stress also affects the mechanism of pectin secretion and conversion, similarly to cold stress [42,43]. The abovementioned alterations morphologically result in smaller and less viable pollen. The reduced performance in terms of pollen germination is associated with deep alterations in the cytoskeleton, Ca^2+^ and ROS localization, and pH profile [41,42,44,45,46]. While the literature mostly deals with plants of agronomic interest, few evidence concerns heat stress impact in natural habitats, addressing the problem of forest tree mortality associated with unusually dry and hot climatic conditions [47,48].

### 2.2. Heavy Metal-Induced Stress

Knowledge of the impact of heavy metals on pollen and the pollen tube is different. Pioneering [49] and more recent studies have shown that heavy metals have significant effects on pollen function; however, general consequences cannot be ruled out due to plant- and metal-specific responsiveness. Studies on *Picea wilsonii* pollen have shown that pollen might show metal-specific effects. In addition, distinct metals can alter the shape of the pollen tube in different ways or induce cytoplasmic aberrations (vacuolization) [50]. For example, Cd has severe effects on tobacco and lily pollen, causing pollen tube deformations and irregularities in Ca^2+^ distribution [51]. Tobacco pollen has also been tested for studying the effects of several heavy metals, including Ni, Fe, Pb, Co, Cd, Hg, Al, Zn and Cu, showing their detrimental effects both on pollen tube germination and growth [52]. Similar effects were also found for *Jatropha curcas* pollen [53], albeit with metal-specific effects. In apple, various heavy metals cause effects proportionally to the dose applied [54] and different apple varieties exhibit specific sensitivities to heavy metals [55]. A dose-effect linearity was also found for apricot and cherry pollen [56]. Nickel shows combined effects, as it does not block germination but prevents pollen tube growth [57]. Lead treatment causes profound alterations in pollen tubes, which show cessation of growth and uneven distribution of cell wall components, most likely by targeting the cytoskeleton [58]. Environmental contamination with arsenic can adversely affect pollen biology. Arsenic species such as arsenite, arsenate, monomethylarsonate, and dimethylarsinate show cytotoxic effects on the pollen tube growth assay of *Nicotiana sylvestris*. The 50% inhibition concentration of pollen tube growth was determined and compared with the LD50 values of the compounds in the literature. Compared to inorganic species, higher cytotoxicity was found for arsenate in the pollen tube growth assay [59]. In kiwi, Cr(III) and Cr(VI) differentially affect the profile of PAs in pollen, with spermidine increasing in pollen tubes treated with Cr(III) but not Cr(VI). In addition, chromium-induced effects are partially counteracted by the accumulation of putrescine, the spermidine precursor, suggesting that tolerance to heavy metals can be achieved by altering the balance of polyamines [60].

### 2.3. Light (UV-B) Stress

UV-B radiation is known to adversely affect plants at several levels, from physiological to morphological. Although pollen has been used as a vector for UV-B-induced mutations [61], there are few studies on the direct effects of UV-B radiation on pollen. UV-B radiation certainly affects pollen to a degree dependent on the targeted species and the plant’s adaptations to specific environments. As shown in soybean, the sensitivity of the male gametophyte to UV-B radiation might be dose-dependent [62]. It is therefore reasonable that pollen of different species (or varieties) exhibits distinct behaviors in relation to UV-B [63]. In some cases, this diverse response results in an enhancement of pollen tube germination by UV-B treatment [64]. An increase in pollen tube germination rate was also observed after brief UV-B exposure, such as in *Nicotiana plumbaginifolia* [65]. On the contrary, in maize, UV-B radiation causes reduced pollen tube germination and growth, probably due to excessive production of ROS and consequent lipid peroxidation [66]. A significant reduction in germination and pollen tube length was also observed in olive after UV-B treatment [67]. How UV-B radiation negatively affects pollen is not fully known. Some evidence points to an involvement of nitric oxide (NO) overproduction and accumulation with deleterious effects on pollen [68]. Most likely, however, there are pathways of damage that are independent of NO. For example, increased production of ROS with associated reduction in germination and pollen tube growth was observed in *Brassica* [69]. UV-B radiation also modulates endogenous hormone levels, as shown in tomato where alteration in the levels of specific hormones following UV-B radiation decreases pollen germination but increases fruit number [70]. During evolution, pollen most likely responded to UV-B treatment by thickening its cell wall to increase the level of protection, as in the case of *Salix polaris* pollen [71]. Studies have shown that a different composition in the sporopollenin layer may have been an important evolutionary trait in pollen because of the different ability to absorb UV-B radiation [72]. For example, the increased synthesis of phenolamides (HCAAs), PAs bound to phenylpropanoids, has been associated with a photoprotective role [73]. It cannot be ruled out that a different shape of flower was an evolutionary trait necessary to protect the pollen [74].

### 2.4. Osmotic Stress

Similar to all plant cells, pollen and pollen tube are characterized by a turgor pressure necessary for growth that must be balanced with the external osmotic pressure. Pollen resistance to osmotic stress has also been used as a parameter for the selection of drought tolerant genotypes [75]. The response to altered osmosis conditions requires pollen to implement compensatory mechanisms based on the use of specific membrane phospholipids (PIP2 and phosphatidic acid) [76,77]. Tolerance to osmotic stress may also require the presence of specific proteins, such as proline-rich proteins, that confer resistance during pollen tube growth [78]. Focusing on the mechanism of pollen tube growth, changes in osmotic pressure revealed how the pollen tube can adapt to new external environments [79] because pollen tubes growing under hyperosmotic or hypoosmotic conditions show changes in cell wall structure, as well as in the tube growth profile [80]. Thereby, the typical periodicity of acidic pectins deposition in tobacco pollen tubes was lost after manipulating osmotic pressure. This was a clear indication that the tube oscillatory growth is affected by external physicochemical conditions and readapts to the new environment. However, it must be emphasized that the change in turgor pressure does not necessarily represent a directional force (i.e., to determine the growth of pollen tubes). Some authors argue that the turgor pressure does not differ within the pollen tube and that it is therefore the overall strength of turgor pressure, but not local differences, that promotes the growth of pollen tubes [81,82].

### 2.5. Nutrient Depletion

Alterations in the oscillatory growth of the pollen tube also occur when pollen is energetically stressed, for example by preventing sucrose metabolism. It is known that oscillatory pollen tube growth does not strictly require respiratory metabolism [83], although NAD(P)H levels fluctuate in relation to pollen tube growth [84]. Because of common molecules and proteins (such as sucrose synthase), metabolism and cell wall synthesis are strongly linked; therefore, energy stress affects cell wall assembly and the growth pattern of the pollen tube. In the absence of sucrose, pollen tubes grow slowly and, most importantly, they lose the regular growth pattern and show no oscillations; in addition, individual components of the oscillator (such as ROS, pH, and calcium ions) show relevant changes [85]. ROS are of particular interest because they are produced during pollen grain rehydration, according to pollen type [86,87,88], and during pollen tube emergence, they accumulate at the tube apex as a result of the metabolic activity of mitochondria and of plasma membrane-associated NAD(P)H oxidases [19]. The latter are supposed to control pollen tube growth rate by preventing growth accelerations and by coordinating the rate of vesicular secretion with the cell wall structure [89]. In addition, ROS interface with other factors such as calcium ions [90] in the apical region of pollen tubes, thereby contributing significantly to regulating the growth pattern of pollen tubes. When pollen tubes grow under energy stress, ROS content is low at the tip [91]; as a result, pollen tubes are still growing but lose their typical growth periodicity.

### 2.6. Cold Stress

Although cold stress responses in plants have been extensively studied, there are few investigations into the effect of cold stress on pollen development and function [92], contrary to heat stress [93]. Cold stress causes several cyto-molecular changes in pollen tubes, including attenuation of the calcium gradient; cells continue to grow but calcium ions are uniformly distributed along the tube axis [43]. This might be explained considering the downregulation of genes encoding for Ca^2+^-binding proteins [92]. Cold stress also causes clear deformities in the typical cylindrical shape of pollen tubes [94]. In the case of cold stress, adequate levels of NO are needed to make the pollen tube more tolerant of harmful conditions; in *Camellia sinensis*, low temperatures induce the synthesis of NO that causes transcriptomic changes in the pollen tube. During pollen tube elongation also Ca^2+^ gradient, vesicle polarized trafficking as well as cell wall biosynthesis are affected through the NO signaling pathway [95]. NO levels are strongly affected by PAs, such that a recent model suggests that PAs regulate pollen tube growth by modulating NO and ROS levels [96]. The network of gene regulation in response to cold stress is complex, with several genes involved, including genes encoding for protein phosphorylases/dephosphorylases, receptors, signal transduction and hormone regulation [92]. Today, what is missing is a further analysis of pollen response to cold stress by mutant analysis and other molecular and cell biological approaches.

## 3. Can Polyamines Ameliorate the Damaging Effect of Stress?

Similar to other plant cells, the ability of pollen to withstand stressful conditions is related to its intrinsic biochemical, physiological, and cytological characteristics, for example the production of HSPs or osmoprotectants [97], as well as the fine-tuning of ROS and Ca^2+^ levels and the ability to build a cell wall suitable for new conditions [98]. PAs have often been associated with the environmental stress response as they interface with various intracellular signaling processes [11,99], such as phosphorylation [100] and cation transport [8].

When plants are subjected to abiotic stress, one possible adaptive response is the increase in PA levels. The literature comprehensively describes changes in PA content in response to altered environmental conditions. For example, during heat and cold stress, PA levels change, and in some cases, PAs might also be redirected to the synthesis of uncommon PAs, the latter being more involved in thermotolerance [101]. Under cold stress, PA rebalance may increase the synthesis of ABA and/or reduce lipid peroxidation indirectly by inhibiting the synthesis of ROS [102]. Tolerance to salt stress is also mediated by PAs, which regulate Na^+^ and K^+^ fluxes [103,104]. Likewise, under salt stress, Pas might counteract drought stress by controlling Ca^2+^ and K^+^ flux, thereby causing stomata to close [105]. Exogenous putrescine can mitigate drought by reducing oxidative stress and increasing the synthesis of endogenous PAs [106]. In the osmotic stress response, PAs likely facilitate and enhance the synthesis of osmoprotectants [107]. PAs are also involved in the response to nutrient deficiencies, such as potassium [108], and in counteracting hypoxic conditions [109]. The protective effect of PAs against abiotic stresses appears therefore evident, but most likely, the effect is not strictly direct or dose dependent; moreover, the protective effect might be limited to specific cells and distinct time frames [110].

The association between susceptibility/tolerance to environmental stress and PA levels is also supported by expression changes of genes encoding for enzymes in the PA synthesis pathway in transgenic plants [111,112,113,114]. Downregulation of the spermidine synthase gene (SPDS) by RNA interference in *Nicotiana tabacum* showed that drought and salt stress can be counteracted by changes in PA content [115]; the mutation enhances tolerance to salinity and drought conditions due to a constant intracellular pool of putrescine (spermidine precursor) and spermine (spermidine product), thus highlighting a different action of the three PAs [116]. This is confirmed by the Arabidopsis mutant defective in spermine synthesis and consequently hypersensitive to drought and salt stress, whose effects can be mitigated by pretreatment with spermine [117]. Overexpression of the SAMDC gene in tobacco led to an accumulation of spermidine and to a concurrent increase in polyamine oxidase activity, which in turn increased the antioxidant response [118]. Similar results were obtained following overexpression of the SAMDC gene in rice [113].

The effect of PAs on pollen tubes is only partially known, and many details are missing. However, the acquired information may help to understand the role of PAs during stress conditions. When applied to pollen tubes, PAs affect several cytological parameters, such as Ca^2+^ and H^+^ flux, ROS accumulation and tube shape [119,120]. Thus, a balanced content and localization of ROS, Ca^2+^ and H^+^ is likely to normalize pollen tube growth. The action of PAs and ROS is interconnected; PAs may play a role in tip growth as precursors of ROS. In *Arabidopsis thaliana*, ROS accumulation at the tip correlates with pollen tube growth. In detail, the ABC transporter AtABCG28, which regulates ROS levels, is localized in secretory vesicles that fuse with the plasma membrane at the pollen tube tip. Deletion of AtABCG28 results in defective pollen tube growth, failure to localize PAs and ROS at the tip of growing pollen tube, and complete male infertility [121]. Spermidine-treated pollen tubes are initially characterized by progressive changes in shape until growth resumes, despite a larger diameter, concomitantly with extensive rearrangements of actin filaments and pH gradient [122].

**Figure 2:**
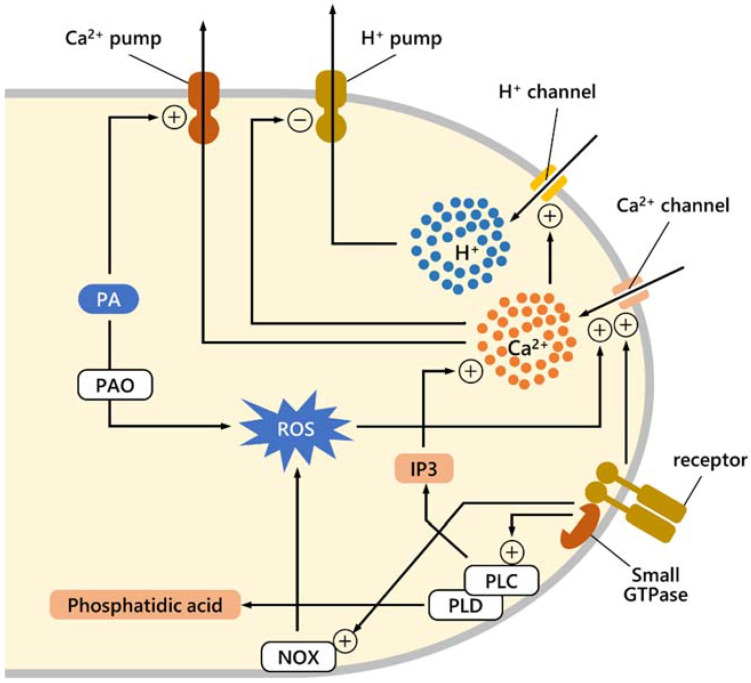
Diagram illustrating some of the mechanisms regulated by PAs underlying Ca^2+^ and proton balance in pollen tube growth. It is supposed that the accumulation of both proton and Ca^2+^ ions, highlighted in the apex, depends on their influx through specific plasma membrane channels. Ion channels are under the control of other effectors; specifically, Ca^2+^ channels are regulated by receptors and small GTPases that mediate external signals. Ca^2+^ accumulation could hypothetically activate proton channels. Ca^2+^ levels are also controlled through another signaling pathway; the GTPase-receptor complex can activate the plasma membrane-associated phospholipase C (PLC) [126], which in turn generates IP3. The latter can stimulate the opening of Ca^2+^ channels. The membrane receptor system most likely also activates the production of ROS through NAD(P)H oxidase; in turn, ROS can affect Ca^2+^ flux. The action of PAs could be implemented in two distinct ways: PAs could activate the efflux of Ca^2+^ in the subapical region, while PAs could contribute to ROS production through the PAO enzyme, thus causing an increase in Ca^2+^ influx. The diagram also shows how the activation of PLC can lead to an increase in Ca^2+^ as mediated by IP_3_ production. Among the membrane phospholipases, phospholipase D (PLD) [127] should also be recalled because it is responsible for the production of phosphatidic acid, a chemical mediator during stressful conditions.

PAs, either produced internally or imported from outside, or directly targeting the surface of pollen tubes, can regulate several molecular processes during pollen tube growth, such as the proper balance of Ca^2+^, protons, and ROS. The mechanism is not known in detail, but currently, available data suggest possible pathways, depicted schematically in Figure 2. In the pollen tube, the exact correlation between Ca^2+^ and H^+^ fluxes and ROS synthesis is not known, although data suggest that Ca^2+^ and ROS may interact. The correlation between Ca^2+^ and H^+^ fluxes is also unknown, although data suggest that increasing Ca^2+^ precedes high growing rates in the pollen tube, whereas H^+^ flux follows fast growth [21]. It is assumed that both Ca^2+^ and H^+^ enter the apical region and are expelled at the subapical region; almost certainly, H^+^ is expelled at the level of the alkaline band, while Ca^2+^ can be actively pumped into organelles. As suggested for other biological systems, if PAs trigger active Ca^2+^ pumping, this will result in dissipation of the cytosolic Ca^2+^ gradient [123]. If Ca^2+^ levels control H^+^ content (either by activating H^+^ influx or inhibiting active H^+^ pumping) and if PAs promote dissipation of the Ca^2+^ gradient, this implies that PAs promote more H^+^ efflux, resulting in dissipation of the H^+^ gradient. The catabolism of PAs produces ROS, which in turn modulates Ca^2+^ [8]. Therefore, PAs could first dissipate the Ca^2+^ gradient, but the subsequent ROS production due to PA catabolism could trigger a new increase in Ca^2+^ levels. Conversely, that PAs can alter ROS levels is well-known and PA metabolism leads to ROS production because of the activity of enzymes such as diamine oxidase (DAO) and PA oxidase (PAO) [96]. Finally, the accumulation of Ca^2+^ levels is also regulated by plasma membrane phospholipases, i.e., phospholipases C (PLC) and phospholipases D (PLD) through distinct pathways. These enzymes modulate cytoskeleton organization [124], are involved in autophagy-mediated cytoplasmic deletion that is necessary for pollen tube emergence [125] and that affect the Ca^2+^ level [126].

The question now is: can PAs play a protective role in pollen against stress? Unfortunately, the current literature reports only a limited amount of useful information. PAs may exert a protective role possibly by regulating the levels of ROS, whose content varies significantly, such as under heat stress [4]. As further evidence, the appropriate dosage of PAs was found to be important in heat-stressed tomato pollen during germination, again underscoring the protective effect of these molecules [128]. Studies in *Prunus* have shown that the protective effect of PAs against stress is dependent on the concentration and type of PAs [129,130], indicating that the beneficial effect of PAs is calibrated on their concentration and that concentrations above a certain threshold have inhibitory effects on pollen tube growth (PAs often have a hormetic effect, and their action involves a dose/response relationship with a biphasic effect, i.e., opposite depending to the dose). Pollen deformities caused by cold stress can also be restored by the addition of spermidine, which allows for normal growth, possibly by recalibrating the pollen tube oscillatory growth. Although cold treatment strongly alters the pH gradient, simultaneous treatment with cold and spermidine causes no apparent damage, and the pollen tubes maintain their normal morphology. The same ameliorative effect is obtained on ROS levels and Ca^2+^ [93]. Further evidence comes from the analysis of transgenic plants. Pollen viability under stress conditions is severely compromised when a key enzyme in PA metabolism (SAMDC) is downregulated [131,132], suggesting that optimal PA levels are required for proper functioning and pollen tolerance capacity.

The action of PAs in counteracting abiotic stresses could also be carried out in concert with enzymatic activities that metabolize PAs; among these is the cross-linking enzyme TGases [87,133], whose activity is enhanced by events that increase cytosolic Ca^2+^, such as rehydration, light, developmental differentiation and stresses as injury, pathogens and induction of programmed cell death (PCD). In some cases, the action of PAs could be mediated by TGase, i.e., pollen cell modeling, ion fluxes regulation and cytoskeleton organization. For more information on the relationship between transglutaminase and pollen tube growth, readers are kindly referred to more specific reviews [134].

One chemical form through which PAs could counteract abiotic stress is phenolamides (HCAAs); these are derived from the binding of PAs to phenylpropanoids, particularly hydrocinnamic acids (HCAs). These molecules have been known since the pioneering studies of Martin-Tanguy and coworkers [135], which led to the identification of HCAAs in the male reproductive organs of maize. HCAs, such as ferulic acid, are bound to the primary and secondary amine groups of PAs (putrescine, spermidine, and/or spermine). HCAAs are pollen specific and synthesized exclusively in the tapetum of developing flowers through the activity of spermidine hydroxycinnamoyltransferase (SHT) [136]. Based on the current data, the requirement of phenylpropanoids for eudicotyledon pollen fertility is unclear, although some evidence (as in the case of SHT-deficient Arabidopsis with an irregular pollen coat) suggests a structural role in the pollen cell wall [136,137]. An interesting function of phenylpropanoids is protection against UV radiation [73]; the binding of all four nitrogen atoms of spermine to HCAAs increases the UV absorbance of a single molecule by about 30% compared to spermidine (which contains only three nitrogen atoms). HCAAs, whether bound to spermidine or spermine, show absorption maxima of 315–330 nm, covering part of the UV-B and UV-A spectrum and thus helping plants cope with this abiotic stress. Finally, HCAAs also play a role as antioxidants and in plant–pollinator interactions. Tris-coumaroyl spermidine, in addition to lipids and flavonols from sunflower pollen, has been reported to stimulate insect feeding [138]. PAs contain nitrogen atoms that could be taken up by insects for their metabolism. If plant–pollinator interactions are stimulated by a cocktail of metabolites that attract pollinators, this could be one reason for the evolutionary success of angiosperms starting with the pioneer *Amborella trichopoda*.

Thus, the role of PAs in mitigating the detrimental effects of abiotic stresses on pollen and fertilization is exerted at several levels, including structural and biochemical. All of this underscores the substantial contribution that PAs can make to plant reproduction, but leaves several questions open, including whether the protective effect is exerted by a specific PA or by an appropriate mix of PAs, which is the optimal concentration of PAs and the best developmental stage for their action.

## 4. Conclusions

Various abiotic stresses in the climate change scenario threaten plant productivity worldwide, while food demand is expected to increase due to population growth and rising incomes. Currently, there are many options available to address this impending food security problem; primarily, priming processes or selection of plant genotypes that are particularly tolerant to stress conditions. Today, the selection process requires several methodological approaches, including phenotyping, marker-assisted selection, but also the identification of genotypes characterized by tolerant pollen. In this perspective, pollen and the pollen tube become markers of stress tolerance. It follows that any methodology or molecular approach that can increase stress tolerance is of great benefit to enable the reproductive process, particularly when it concerns plants of economic interest. PAs are one of the possible targets of these strategies. As highlighted in this review, the synthesis and accumulation of PAs, indicators of plant well-being or stress-relieving agents, can be beneficial for pollen tube growth. Although the role of PA metabolism for pollen abiotic stress tolerance is only beginning to be understood, pollen capable of accumulating and/or biosynthesizing adequate amounts of specific PAs at a given stage of development may be more capable to promote and support plant reproduction under stressful conditions. Many efforts are still needed to understand in detail the molecular mechanism of the protective role of PAs in tolerance to pollen abiotic stress. High throughput analyses including microarrays, transcriptomics, metabolomics, and reverse genetics approaches would be helpful to elucidate the involvement of pollen PAs in stress perception and response. In addition, three-dimensional structural studies of PA interaction partners would also be of significant help in confirming the stress resistance mechanisms hypothesized here. Improving plant tolerance and crop production could also be achieved by exogenous PAs during the most susceptible phases of plant reproduction, as highlighted in this review.

## Data Availability

Not applicable.
